# Left atrial reservoir strain as a predictor of cardiac dysfunction in a murine model of pressure overload

**DOI:** 10.1111/apha.14277

**Published:** 2025-01-16

**Authors:** John P. Salvas, Thomas Moore‐Morris, Craig J. Goergen, Pierre Sicard

**Affiliations:** ^1^ Weldon School of Biomedical Engineering Purdue University West Lafayette Indiana USA; ^2^ Indiana University School of Medicine Indianapolis Indiana USA; ^3^ Institut de Génomique Fonctionnelle, University of Montpellier, CNRS, INSERM Montpellier France; ^4^ PhyMedExp, IPAM/Biocampus, University of Montpellier, INSERM, CNRS. Montpellier France

**Keywords:** heart failure, high‐resolution ultrasound, left atrial strain, prediction

## Abstract

**Aim:**

Left atrial (LA) strain is emerging as a valuable metric for evaluating cardiac function, particularly under pathological conditions such as pressure overload. This preclinical study investigates the predictive utility of LA strain on cardiac function in a murine model subjected to pressure overload, mimicking pathologies such as hypertension and aortic stenosis.

**Methods:**

High‐resolution ultrasound was performed in a cohort of mice (*n* = 16) to evaluate left atrial and left ventricular function at baseline and 2 and 4 weeks after transverse aortic constriction (TAC). Acute adaptations in cardiac function were assessed in a subgroup of mice (*n* = 10) with 3 days post‐TAC imaging.

**Results:**

We report an increase in LA max volume from 11.0 ± 4.3 μL at baseline to 26.7 ± 16.7 μL at 4 weeks (*p* = 0.002) and a decrease in LA reservoir strain from 20.8 ± 5.4% at baseline to 10.2 ± 6.9% at 4 weeks (*p* = 0.001). In the acute phase, LA strain dysfunction was present at 3 days (*p* < 0.001), prior to alterations in LA volume (*p* = 0.856) or left ventricular (LV) ejection fraction (*p* = 0.120). LA reservoir strain correlated with key indicators of cardiac performance including left ventricular (LV) ejection fraction (*r* = 0.541, *p* < 0.001), longitudinal strain (*r* = −0.637, *p* < 0.001), and strain rate (*r* = 0.378, *p* = 0.007). Furthermore, markers of atrial structure and function including LA max volume (AUC = 0.813, *p =* 0.003), ejection fraction (AUC = 0.853, *p* = 0.001), and strain (AUC = 0.884, *p* < 0.001) all predicted LV dysfunction.

**Conclusion:**

LA strain and function assessments provide a reliable, non‐invasive method for the early detection and prediction of cardiac dysfunction in a model of pressure overload.

## 1 INTRODUCTION

Left atrial (LA) remodeling is common in various cardiovascular conditions, including atrial fibrillation, diastolic dysfunction, and heart failure.^1^, ^2^ Monitoring LA evolution during cardiovascular disease has emerged as a valuable biomarker for risk stratification and ongoing risk assessment.^3^, ^4^ Beyond just measuring size, assessing LA functional dynamics provides critical insight into the heart's structural and functional adaptations, particularly in the characterization of left ventricular (LV) diastolic function.^1^ This is especially important in heart failure where LA dysfunction can significantly affect overall cardiac output.^5^ In particular, pressure‐overload conditions, such as those induced by uncontrolled hypertension or aortic stenosis, place a significant burden on the LV, which in turn impacts the LA. As the LV faces increased afterload, the LA must exert greater effort to maintain efficient blood flow and support ventricular filling. This increased workload on the LA can lead to chronic alterations in its structure and function.^6^, ^7^ Recently, LA strain, a measure of the deformation of the LA myocardium during the cardiac cycle, has gained attention for its potential to provide deeper insights into atrial function.^8^ This includes different phases of LA strain, such as the reservoir phase, during which the left atrium fills and stretches, offering valuable information on atrial compliance and function. In clinical settings, early changes in LA mechanics are often associated with the severity of heart failure and other adverse cardiovascular outcomes.^9^, ^10^ However, despite its clinical relevance, the measurement of LA strain and its implications in murine models of heart failure remain insufficiently studied.^11^, ^12^, ^13^ Here, we used advanced echocardiographic techniques to precisely quantify LA strain and examined its relationship with other indices of LV dysfunction following acute and chronic transverse aortic constriction (TAC). Overall, our findings suggest that acute altered LA reservoir strain is predictive of cardiac dysfunction in pressure overload.

## 2 RESULTS

### 2.1 Ultrasound reveals progressive left atrial remodeling following TAC

**FIGURE 1:**
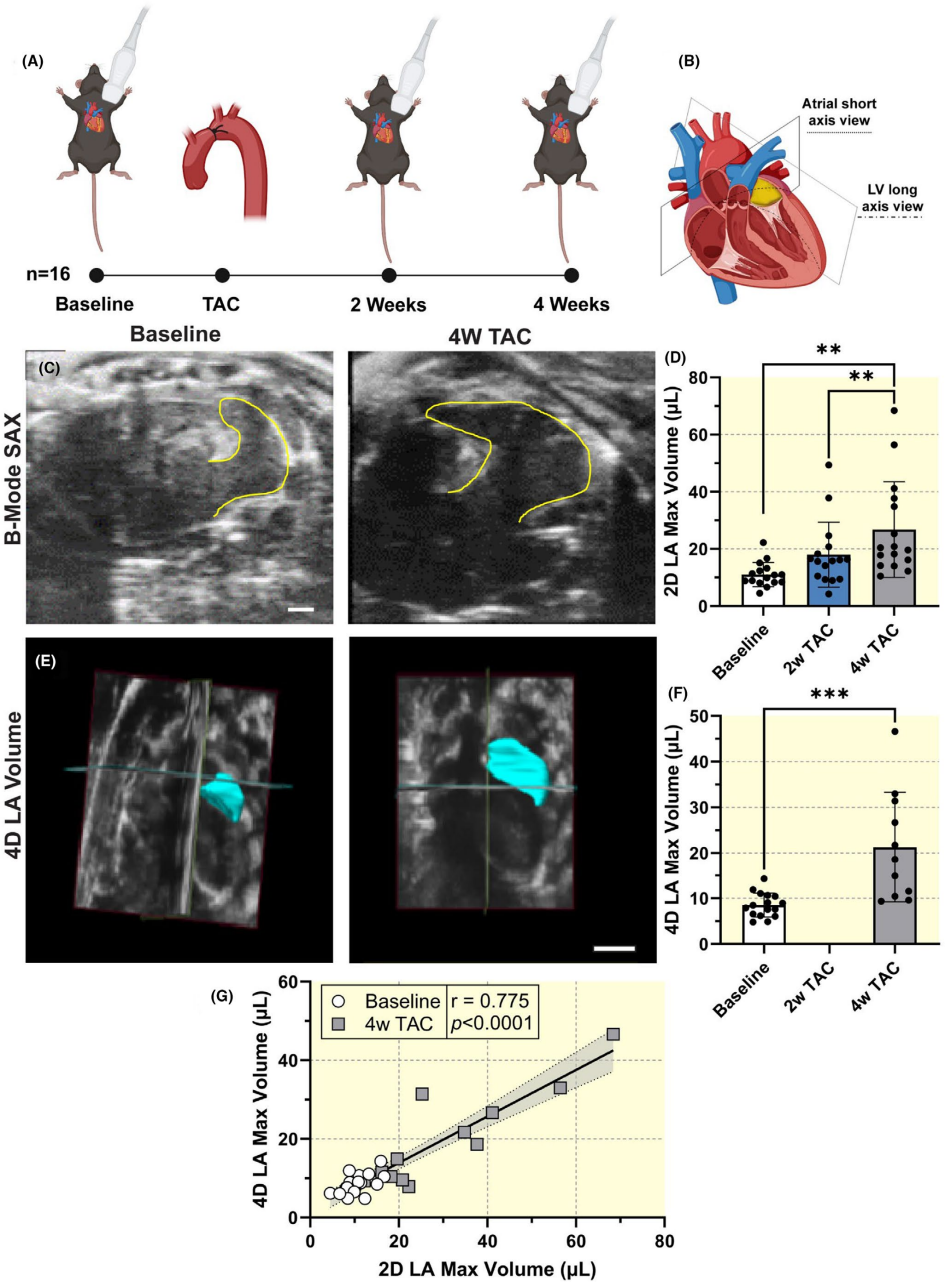
Experimental design and left atrial (LA) volumes. (A) Study design and timeline. (B) Representation of left atrial short‐axis and left ventricular long‐axis imaging. (C) Representative short‐axis ultrasound images of the left atrium at baseline and 4‐week post‐TAC procedure. Scale bar represents 1 mm. (D) Left atrial maximum volume. Sample size *n* = 16. Results expressed as mean ± SD. Statistical comparison conducted with one‐way repeated measures ANOVA with Tukey's post hoc multiple comparisons tests. (E) Representative four‐dimensional (4D) LA volumes at baseline and 4‐week post‐TAC. Scale bar represents 3 mm. (F) 4D LA maximum volume. Sample size *n* = 16 for baseline and *n* = 11 for 4‐week post‐TAC. Results expressed as mean ± SD. Statistical comparison conducted with non‐paired studentized *t*‐test. ** and *** indicate *p*<0.01 and *p*<0.001, respectively. (G) Spearman nonparametric correlation between 4D and 2D LA max volumes. Sample size *n* = 27. Correlation coefficient and corresponding *p*‐value displayed.

High‐resolution ultrasound was used to assess LA anatomical and functional activity 2 and 4 weeks after TAC (Figure 1A). We performed 2D scans in the short‐axis view at the base of the heart to obtain a clear view of the left atrium (Figure 1B,C). Consistent with previous reports,^14^ the LA showed signs of early structural remodeling due to cardiac pressure overload, indicated by a progressive increase in LA maximum volume (baseline: 11.0 ± 4.3 μL, 2W TAC: 18.0 ± 11.4 μL; 4W TAC: 26.7 ± 16.7 μL, *p* = 0.002; Figure 1D). In addition, four‐dimensional (4D) ultrasound assessments of LA max volume showed a similar significant increase in max volume from baseline to 4‐week post‐TAC (*p <* 0.001) and correlated with 2D volume estimations (*r* = 0.775, *p <* 0.0001; Figure 1E–G).

### 2.2 Pressure overload leads to sustained decline in left atrial strain assessed by speckle tracking

**FIGURE 2:**
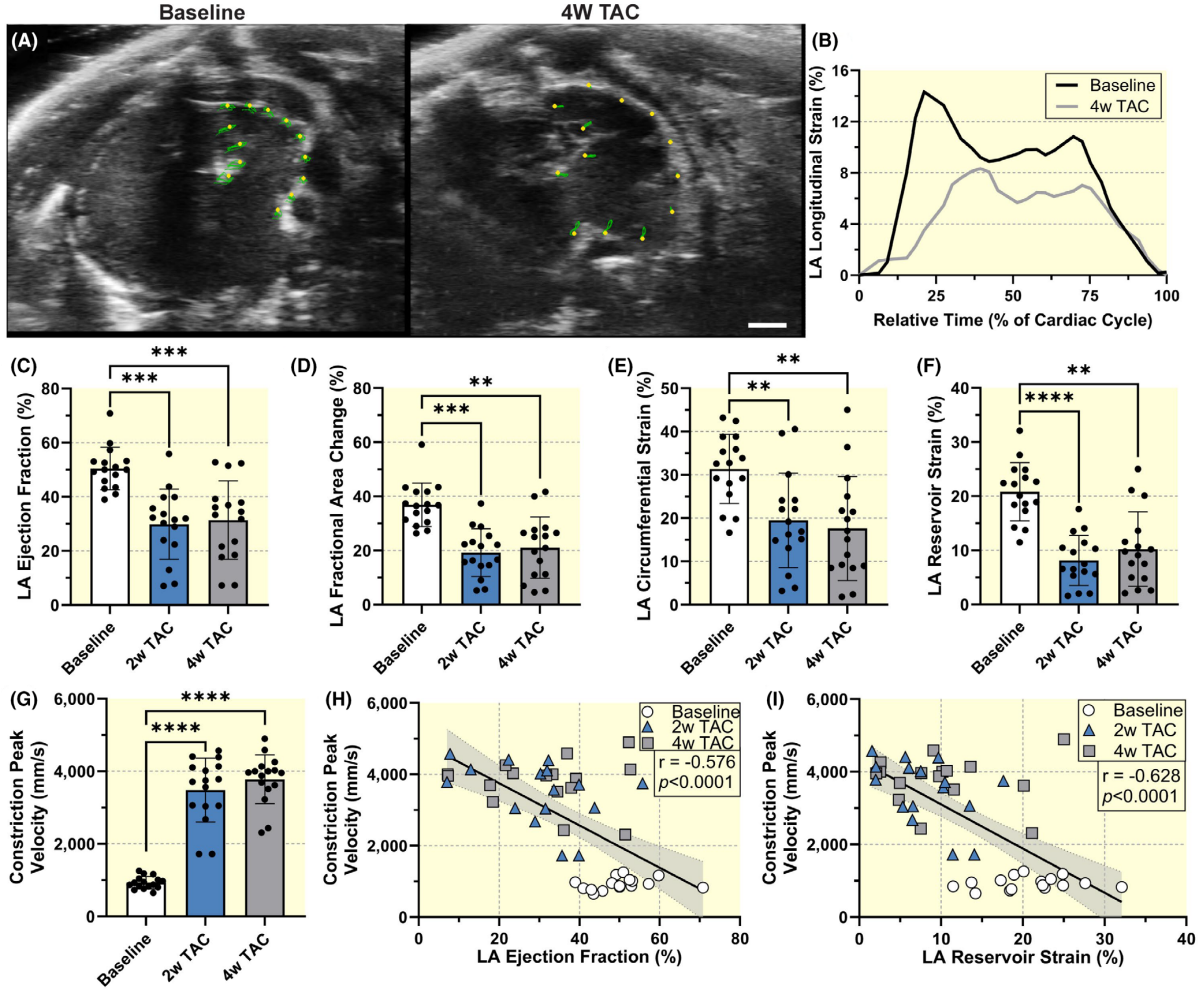
Speckle‐tracking analysis of left atrial function in a pressure overload model. (A) Representative baseline and 4‐week post‐TAC example short‐axis views of the left atrium in the VevoStrain 2.0 software with yellow points representing the atrial myocardial wall tracking and green lines representing the myocardial motion over the cardiac cycle. Scale bar represents 1 mm. (B) Representative global longitudinal strain curve for the left atrium at baseline and 4‐week post‐TAC. Left atrial (C) ejection fraction, (D) fractional area change, (E) circumferential strain, and (F) reservoir strain. (G) Aortic constriction peak blood flow velocity. Sample size *n* = 16 for each timepoint. Results expressed as mean ± SD. Statistical comparisons conducted with one‐way repeated measures ANOVA with Tukey's post hoc multiple comparisons tests. **, ***, and **** indicate *p*<0.01, *p*<0.001, and *p*<0.0001, respectively. Spearman correlation of constriction peak flow velocities with left atrial (H) ejection fraction and (I) reservoir strain from all timepoints. Sample size *n* = 48. Correlation coefficients and corresponding *p*‐values displayed.

Next, we tested the hypothesis that pressure overload decreases LA function over time. Using a specific 2D basal short‐axis view and speckle tracking analysis allows for the calculation of LA function parameters including ejection fraction and strain (Figure 2A,B). LA ejection fraction (EF) significantly decreased from 50.5 ± 7.8% at baseline to 29.9 ± 12.9% at 2‐weeks post‐TAC (*p* < 0.001) and remained consistently decreased at 4 weeks (31.4 ± 14.5, *p* = 0.928; Figure 2C). Additionally, LA fractional area changes and the circumferential strain showed similar trends with significant decreases from baseline to 2 weeks and non‐significant changes from 2‐ to 4‐week post‐TAC (Figure 2D,E). LA reservoir strain was 20.8 ± 5.4% at baseline and significantly decreased to 8.1 ± 4.6% at 2 weeks (*p* < 0.0001) and remained consistently decreased at 4 weeks compared to 2 weeks (10.2 ± 6.9%, *p* = 0.569; Figure 2F). LA contractile and conduit strains also showed significant reductions 2‐ and 4‐week post‐TAC (Figure S1A–C). Next, we examined the relationship between left atrial (LA) function and the degree of pressure overload, quantified by aortic constriction peak flow velocity. At baseline, the peak flow velocity was 932 ± 170 mm/s and increased significantly to 3482 ± 880 mm/s at 2 weeks (*p* < 0.0001), remaining consistently elevated at 4 weeks (*p* = 0.095), confirming a successful transverse aortic constriction (TAC) procedure (Figure 2G). Both LA ejection fraction (*r* = −0.576, *p* < 0.0001) and reservoir strain (*r* = −0.628, *p* < 0.0001) were inversely correlated with the peak flow velocity (Figure 2H,I). In contrast, heart rate (Figure S2A–C) did not correlate with cardiac function.

### 2.3 Structural remodeling drives left atrial functional adaptations

**FIGURE 3:**
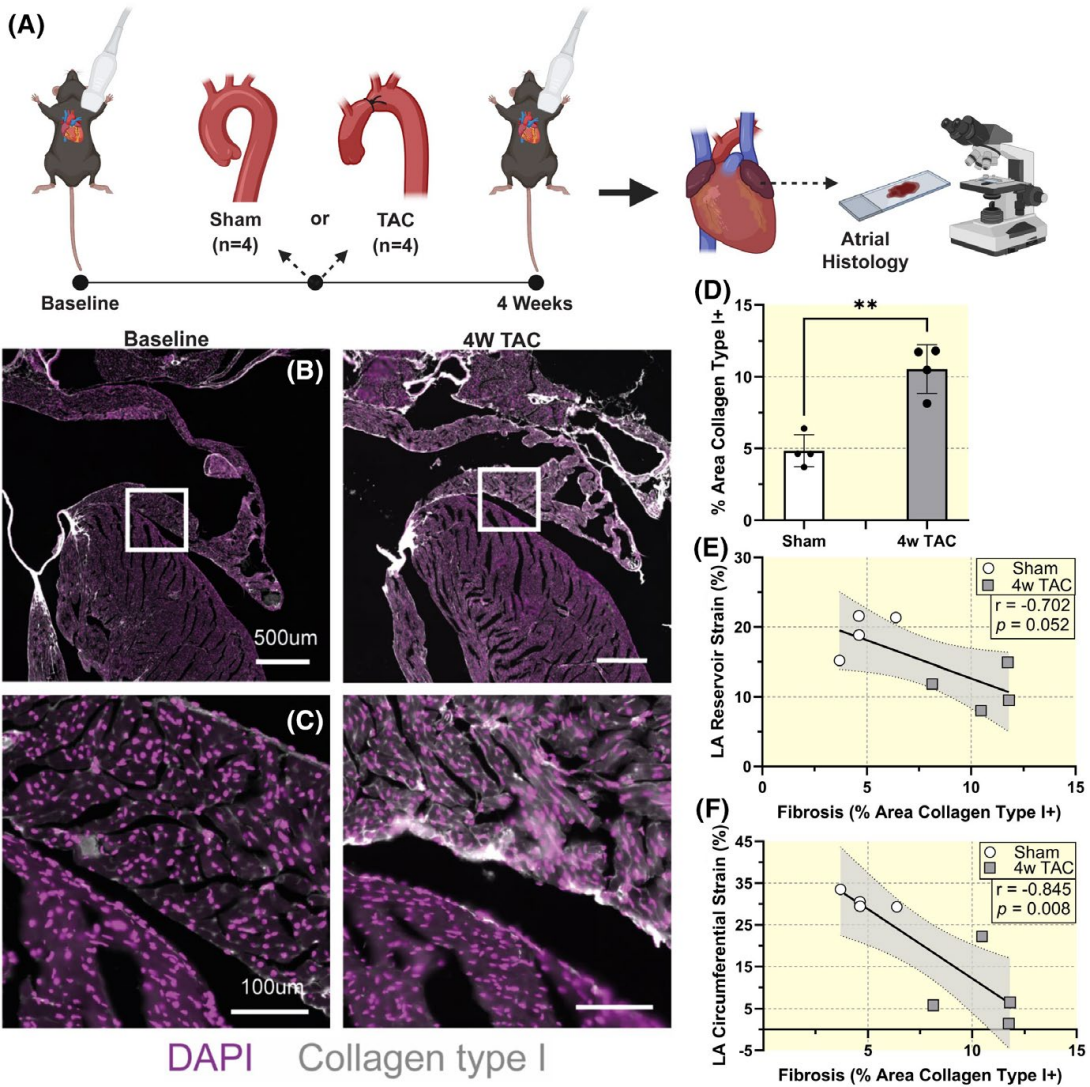
Left Atrial histology. (A) Histologic subgroup study design with sham (*n* = 4) and TAC (*n* = 4) procedure groups. (B) Representative histologic cross sections of the left atrium and atrial appendage at baseline and 4‐week post‐TAC. (C) Insets showing higher magnification histologic cross sections of the left atrium at baseline and 4‐week post‐TAC. Immunofluorescence staining shows collagen type I+ (grayscale) and DAPI/nuclei (violet). (D) Left atrial fibrosis quantification in sham and 4‐week post‐TAC animals. Sample size *n* = 4 for each group. Results expressed as mean ± SD. Statistical comparison conducted with non‐paired studentized *t*‐test. ** indicates *p*<0.01. Pearson correlation of left atrial (E) reservoir strain and (F) circumferential strain with fibrosis, as quantified by % area of collagen type I+. Sample size *n* = 8. Correlation coefficients and corresponding *p*‐values displayed.

In a histologic cohort, sham (*n* = 4) and TAC (*n* = 4) animals underwent baseline and 4‐week post‐procedure cardiac imaging followed by histologic assessment of the left atrial myocardium (Figure 3A–C). There was increased atrial interstitial fibrosis after TAC, as evidenced by increased collagen type I+ area (sham: 4.8 ± 1.1%, 4w TAC: 10.5 ± 1.7%; *p* = 0.001; Figure 3D). Remarkably, the level of fibrosis correlated with LA reservoir (*r* = −0.702, *p* = 0.052) and circumferential strain (*r* = −0.845, *p* = 0.008; Figure 3E,F).

### 2.4 Left ventricular dysfunction after TAC

**FIGURE 4:**
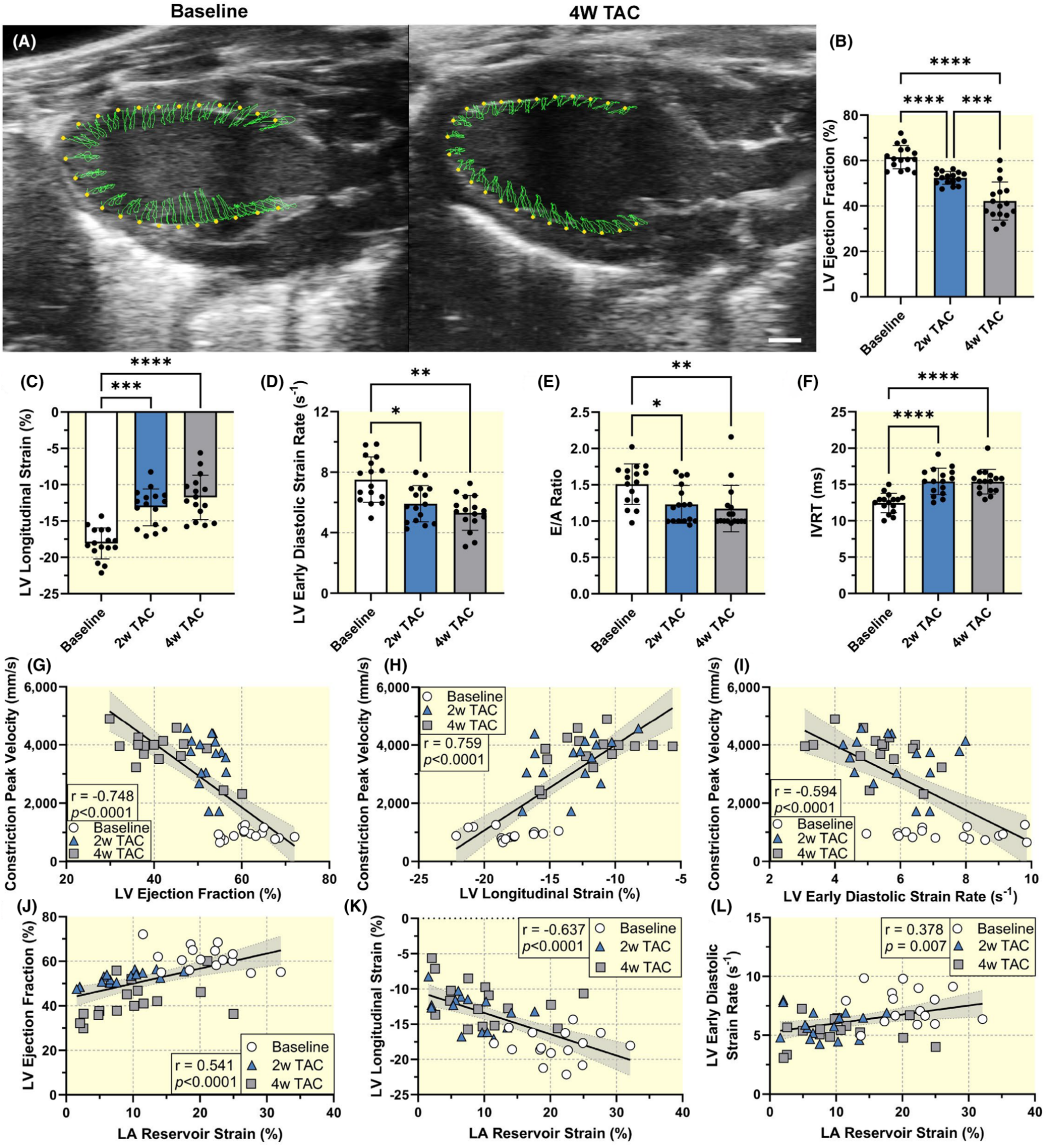
Left ventricular dysfunction post‐TAC. (A) Representative baseline and 4 weeks post‐TAC parasternal long‐axis views of the left ventricle in the VevoStrain 2.0 software with yellow points representing the ventricular myocardial wall tracking and green lines representing the myocardial motion over the cardiac cycle. Scale bar represents 1 mm. Left ventricular (B) ejection fraction, (C) longitudinal strain, (D) early diastolic strain rate, (E) E/A ratio, and (F) isovolumic relaxation time (IVRT). Sample size *n* = 16 for each timepoint. Results expressed as mean ± SD. Statistical comparisons conducted with one‐way repeated measures ANOVA with Tukey's post hoc multiple comparisons tests.*, **, ***, and **** indicate *p*<0.05, *p*<0.01, *p*<0.001, and *p*<0.0001, respectively. Spearman correlation of constriction peak flow velocities with left ventricular (G) ejection fraction, (H) longitudinal strain, and (I) early diastolic strain rate from all timepoints. Pearson correlation of left ventricular (J) ejection fraction, (K) longitudinal strain and (L) early diastolic strain rate versus left atrial reservoir strain. Sample size *n* = 48 which includes measurements from all timepoints. Correlation coefficients and corresponding *p*‐values displayed.

We then evaluated how LA strain dysfunction is associated with heart failure development. After TAC, animals displayed signs of cardiac decompensation indicated by significant reductions in LV contractile systolic and diastolic function (Figure 4A–F). As expected, LV EF was 61.5 ± 5.2% at baseline and progressively decreased from 52.4 ± 2.8% at 2 weeks to 42.2 ± 8.4% at 4 weeks (*p* < 0.001) while LV longitudinal strain was −18.1 ± 2.1% at baseline and remained consistently decreased at −13.1 ± 2.6% and −11.7 ± 3.1% (*p* = 0.238) at 2‐ and 4‐week post‐TAC, respectively. LV early diastolic strain rate followed a similar trend with a significant reduction from baseline to 2 weeks (*p* = 0.014) but no further significant change at 4 weeks (*p* = 0.287). The E/A ratio was 1.51 ± 0.28 at baseline and decreased to 1.23 ± 0.26 2‐weeks post‐TAC (*p* = 0.022) and was consistently decreased 4‐weeks post‐TAC (*p* = 0.838; Figure 4E). The isovolumic relaxation time (IVRT) was 12.5 ± 1.3 ms at baseline and increased to 15.4 ± 1.8 ms 2‐weeks post‐TAC (*p* < 0.0001) and was consistently increased 4‐weeks post‐TAC (*p* = 0.998; Figure 4F). Thus, we confirmed TAC induces left ventricular systolic and diastolic dysfunction. We then assessed whether LV parameters correlated with constriction peak flow velocities. LV ejection fraction (*r* = −0.748, *p <* 0.0001), longitudinal strain (*r* = 0.759, *p <* 0.0001), and early diastolic strain rate (*r* = −0.594, *p <* 0.0001) measurements were all significantly associated with peak flow velocity (Figure 4G–I). Both E/A ratio and IVRT also correlated with constriction peak flow velocities (*p* < 0.001; Figure S3). Importantly, LA strain measurements were significantly correlated with ventricular systolic function (ejection fraction: *r* = 0.541, *p* < 0.0001; strain: *r* = −0.637, *p* < 0.0001) and diastolic function over time (early diastolic strain rate: *r* = 0.378, *p* = 0.007; Figure 3D–G). Moreover, multiple significant correlations were observed between LA and LV function parameters (Figure S3A). Thus, we demonstrate that LA strain correlates with LV dysfunction.

### 2.5 Early left atrial strain dysfunction predicts late left ventricular failure

**FIGURE 5:**
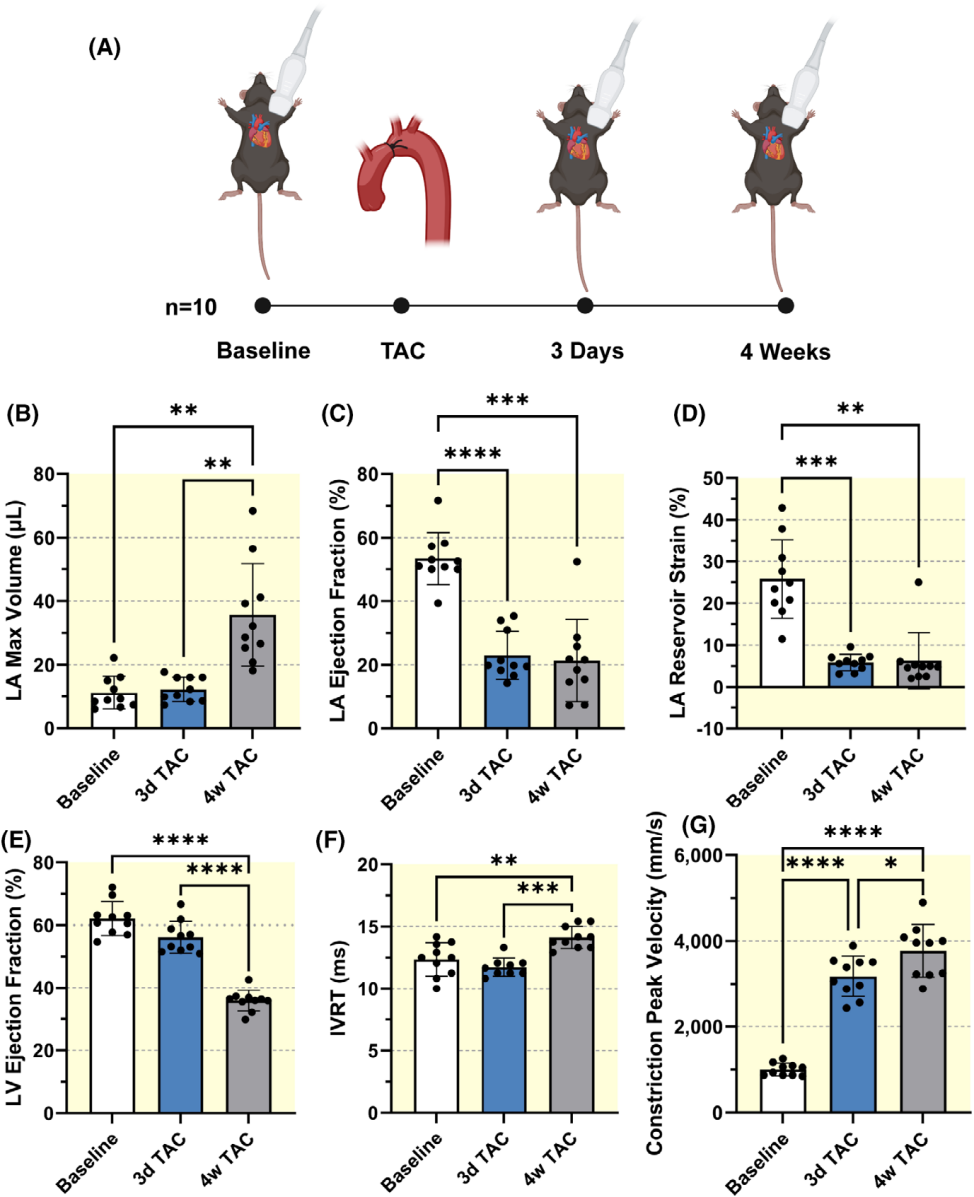
Acute left atrial dysfunction after TAC. (A) Subgroup study design and timeline. Left atrial (B) maximum volume, (C) ejection fraction, and (D) reservoir strain. (E) Left ventricular ejection fraction and (F) isovolumic relaxation time (IVRT), *n* = 9 at 3 days. (G) Constriction peak blood flow velocity. Sample size *n* = 10 for each timepoint, unless otherwise noted. Results expressed as mean ± SD. Statistical comparisons conducted with one‐way repeated measures ANOVA with Tukey's post hoc multiple comparisons tests. Mixed‐effects model fit for IVRT to account for unequal sample sizes. *, **, ***, and **** indicate *p*<0.05, *p*<0.01, *p*<0.001, and *p*<0.0001, respectively.

To further evaluate the acute phase of LA remodeling after TAC, we performed a subgroup analysis with imaging at baseline, 3 days post‐TAC, and 4‐weeks post‐TAC (Figure 5A). Surprisingly, LA max volume remained consistent from baseline to 3 days (baseline: 11.2 ± 5.2 μL, 3 days: 12.3 ± 3.9 μL, *p* = 0.856) before significantly increasing to 35.7 ± 16.1 μL at 4‐weeks post‐TAC (*p* = 0.001; Figure 5B). In contrast, LA ejection fraction (baseline: 53.4 ± 8.2%, 3 days: 23.0 ± 7.5%, *p* < 0.001) and reservoir strain (baseline: 25.8 ± 9.4%, 3 days: 5.9 ± 2.0%, *p* < 0.001) dysfunction were already present 3 days after induced pressure overload (Figure 5C,D). LA contractile and conduit strain also showed significant reductions 3‐days and 4‐weeks post‐TAC (Figure S1D,E). Furthermore, LV EF was 62.2 ± 5.4% at baseline and remained stable at 56.2 ± 5.1% at 3 days (*p* = 0.120) before significantly decreasing to 35.9 ± 3.3% at 4 weeks (Figure 5E). IVRT was 12.4 ± 1.3 ms at baseline and remained stable at 11.7 ± 0.7 ms 3‐day post‐TAC (*p* = 0.606) before significantly increasing to 14.1 ± 0.9 ms 4‐weeks post‐TAC (Figure 5F). Aortic constriction peak flow velocity was increased in magnitude at 3 days (*p* < 0.001), demonstrating successful TAC (Figure 5G).

**FIGURE 6:**
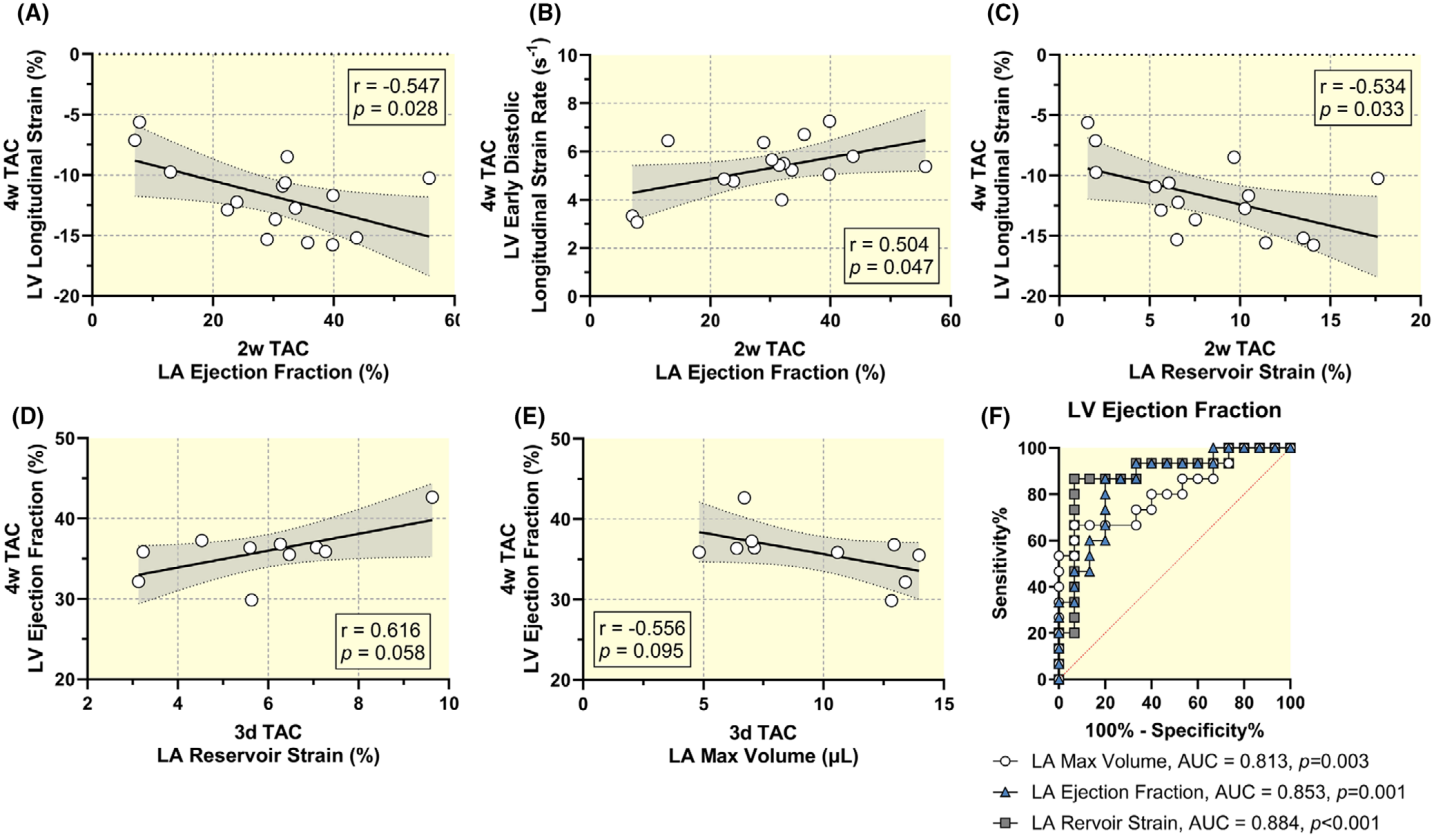
Early left atrial dysfunction predicts ventricular dysfunction. Pearson correlation of 2‐week post‐TAC LA ejection fraction with (A) 4‐week post‐TAC LV longitudinal strain and (B) early diastolic strain rate. Sample size *n* = 16. (C) Pearson correlation of 4‐week post‐TAC LV longitudinal strain with 2‐week post‐TAC LA reservoir strain. Sample size *n* = 16. Correlation of 4‐week post‐TAC LV ejection fraction with 3‐day post‐TAC (D) LA reservoir strain and (E) max volume. Sample size *n* = 10. (F) ROC analysis in the subgroup using left atrial parameters (max volume: AUC = 0.813, *p* = 0.003; ejection fraction: AUC = 0.853, *p* = 0.001; reservoir strain: AUC = 0.884, *p* < 0.001) to predict ventricular ejection fraction above (*n* = 15) or below (*n* = 15) the median (LVEF = 54.0%).

Given these findings, we investigated the hypothesis that early assessment of LA function could predict later LV dysfunction by examining the correlation between LA function at 2‐weeks post‐TAC and LV function at 4‐weeks post‐TAC. (Figure 6A–C). LA EF significantly correlated with both LV longitudinal strain (*r* = −0.547, *p =* 0.028) and strain rate (*r* = 0.504, *p* = 0.047). Early LA reservoir strain also correlated with LV longitudinal strain (*r* = −0.534, *p* = 0.033) at 4 weeks. In the subgroup, we correlated LA function 3 days after TAC and LV function 4 weeks after TAC. Early LA reservoir strain dysfunction correlated with late ventricular ejection fraction (*r* = 0.616, *p* = 0.058). LA max volume was also weakly associated with LV ejection fraction at 4 weeks despite no change from baseline to 3‐day TAC (*r* = −0.556, *p* = 0.095; Figure 6D,E). Further correlations between early LA and late LV dysfunction are shown in Figures S3B and S4B. Significant correlations were found between additional LA and LV parameters such as LA reservoir strain and LV circumferential strain, suggesting that early LA dysfunction quantified by LA strain may predict late LV dysfunction.

Finally, we assessed whether LA dysfunction could serve as a valuable imaging biomarker for distinguishing between low and high LV ejection fraction (EF) groups, using a median LV EF cutoff of 54.0%. The ROC curves illustrate the diagnostic performance of LA max volume (AUC = 0.813), LA EF (AUC = 0.853), and LA reservoir strain (AUC = 0.884) in differentiating between TAC mice with varying levels of LV EF in the subgroup. All parameters of the LA function were able to significantly discriminate between low and high LVEF with *p* < 0.003 (Figure 6F). A further principal component analysis (PCA) of the main cohort can be found in Figure S5 which highlights important variables for discrimination between low and high LV EF groups.

## 3 DISCUSSION

Overall, we report an original approach for LA strain analysis and show that LA dysfunction preceded LV failure. The correlation observed between LA strain and subsequent LV loss of function suggests that the former is a predictor of heart failure. Historically, the LA has been recognized as a crucial buffer chamber that helps maintain cardiac output and normal LV diastolic pressure.^15^, ^16^ More recently, measuring LA structure and function has gained emphasis in heart failure management, as LA dysfunction can exacerbate symptoms and is associated with worse outcomes in heart failure patients.^5^, ^17^ Despite its importance, atrial evaluation is underrepresented in preclinical studies. LA dimensions are a recognized biomarker for studying LV dysfunction across various heart failure models.^6^, ^18^ However, imaging of the LA in murine models of heart failure remains challenging due to the lack of standardized criteria and consensus, as well as the specific geometry and complex function of the LA.^11^, ^18^, ^19^ Recently, Matsiukevich et al. implemented a modified parasternal long‐axis approach and used a disk summation method for LA volume and automated speckle‐tracking for LA strain estimation.^13^ Alternative studies by Zhang et al. have also followed this approach for LA imaging and strain quantification,^11^ while work by Perike et al. implemented an oblique short‐axis imaging approach similar to our methods.^12^ Despite the lack of consensus approach to LA imaging, reported baseline peak reservoir strain and LA ejection fraction values are similar across studies.^11^, ^12^, ^13^ In our study, we demonstrate the feasibility of assessing murine LA dimension using high‐resolution echocardiography at both early and late stages of pressure overload. Our modified short‐axis approach allowed for clear localization of the LA between the LV base and the pulmonary artery. This orientation allowed for the precise detection of complex LA geometry and facilitated the estimation of 2D volumes, which were subsequently validated using 4D imaging. Using this technique and in association with histology we confirmed that pressure overload induces structural remodeling in the LA.^6^, ^14^, ^20^

Anatomically, the LA has a much thinner layer of cardiomyocytes compared to the LV, making it more sensitive to pressure overload and impacting its function. Utilizing specialized speckle‐tracking software, we successfully evaluated LA strain trajectories in a murine model of pressure overload. We demonstrated that atrial strain dysfunction and increased LA volume correlate with subsequent LV dysfunction. Furthermore, we hypothesized that LA strain could serve as an early biomarker for heart failure development in this model of transverse aortic constriction. Consistent with previous clinical evidence,^10^, ^21^ we found that LA dysfunction precedes LV dysfunction. Importantly, using an ROC curve analysis and PCA, we found that early LA structural and functional modifications can predict the trajectory of LV function following pressure overload. These findings are consistent with recent clinical studies,^22^, ^23^, ^24^ which reported that LA dysfunction is highly predictive of cardiovascular outcomes and could be employed as a biomarker to improve risk stratification.

Previous studies have indicated that LA functional impairment can be detected even in the absence of atrial structural changes, suggesting it may be an earlier indicator of cardiac damage, particularly in patients with aortic stenosis.^25^ Furthermore, some clinical studies have suggested that LA strain dysfunction may precede LA volume adaptations.^26^, ^27^ Our findings confirm that LA strain dysfunction occurs in the acute phase of pressure overload, preceding abnormal LA geometry, suggesting LA strain is a more sensitive biomarker than volume alone.

While the predictive utility of LA strain shows promise, there are limitations. While atrial fibrillation is an independent predictor of heart failure, LA strain is also decreased in patients with atrial fibrillation. It is not, however, known whether the predictive value stems from the dyssynchrony of atrial fibrillation or atrial remodeling and the subsequent decreased strain.^28^ Further, while numerous studies have shown that LA reservoir strain is associated with diastolic function as assessed by LV filling pressure in heart failure with reduced EF^28^, ^29^, ^30^ and to a lesser extent in heart failure with preserved EF,^31^ LA strain is not a direct index of LV function.^28^

The molecular mechanisms underlying structural and functional alterations in the LA are not well understood. In a recent study by Yamaguchi et al., transcriptomic data from a model of pressure overload, 2 weeks post‐intervention, revealed that the most significantly upregulated or downregulated biological processes in the LA involve pathways associated with collagen deposition, hypertrophic and dilated cardiomyopathy, oxidative phosphorylation, and metabolic processes.^14^ However, the molecular profile of acute LA modifications during the early phase of pressure overload remains unknown. As the LA is highly sensitive to pathologic stimuli such as pressure overload, investigating how gene responses correlate with acute LA strain dysfunction could reveal novel biomarkers and be a crucial area of research in the future. This method offers a promising technique for more accurate and reproducible assessments of LA function in preclinical studies.

### 3.1 Limitations

In our study, LA function was evaluated only in two dimensions, which may have led to under‐ or overestimation of LA strain. However, while 2D volume estimates were validated with 4D volume measurements, assessing LA conduit function in 2D remained challenging due to the difficulty in distinguishing between the reservoir and contractile phases. Additionally, the LA and left atrial appendage (LAA) were considered one chamber for analysis due to the relatively large size and contribution of the LAA to overall LA function in mice.^19^ In humans, the LAA plays a smaller role in overall LA function and thus analyses of the distinct global and regional function of the LA and LAA are warranted. Future work could include development of advanced 4D imaging and analysis techniques to accurately evaluate LA anatomy and function throughout the cardiac cycle similar to advanced 4D left ventricular analysis techniques.^32^

## 4 MATERIALS AND METHODS

### 4.1 Animal model

Experiments were performed in concordance with the European Directive (2010/63/EU) and French laws governing laboratory animal use. The study was approved by the institutional ethics committee (#2023020112386679v3). We used 8‐week‐old C57BL/6J mice with *n* = 16 in the main cohort and *n* = 5 in the subgroup. The animal habitat was maintained at a constant 21 ± 1°C and 55 ± 1% humidity. Mice had access to food and water ad libitum.

High‐resolution ultrasound imaging was performed on a cohort of mice (*n* = 16) at baseline, 2‐, and 4‐weeks post‐transverse aortic constriction (TAC) (Figure 1A). A subgroup of mice (*n* = 5) and randomly assigned mice from the original cohort (*n* = 5) underwent additional evaluation to elucidate the acute effects of TAC on the left atrium. High‐resolution ultrasound was performed in the subgroup at baseline, 3‐days, and 4‐weeks post‐TAC (Figure 5A). Five of the original cohort mice were imaged 3‐days post‐TAC in addition to the baseline, 2‐, and 4‐week TAC timepoints.

### 4.2 Transverse aortic constriction

Mice were anesthetized with 2.5% isoflurane at 1.0 mL/min and a heating pad (Temperature Control Unit HB101/2, Panlab, USA) was used to maintain body temperature at 37°C. Buprenorphine (1 mg/kg) was administered by subcutaneous injection. The animals were secured to a sterile surgical stage in a supine position, and a small incision was made in the neck to visualize the trachea for intubation. The animals were intubated and placed on a ventilator (MiniVent Type 845, Hugo Sachs Elektronik, Germany) with a mixture of room air and 2.5% isoflurane with a respiratory rate of 140 breaths/min and a tidal volume of 225 μL. The ventral thorax hair was removed, and the skin was sterilized with betadine.

As previously described, a closed‐chest model was used to induce the transverse aortic constriction (TAC).^33^, ^34^ Briefly, a 0.5–1.0 cm transverse incision was made in the left 2nd intercostal space. An electrosurgical unit was used to dissect the pectoralis muscle. A blunt dissection technique was then used to visualize the aortic arch. A small homemade J‐hook wire was used to isolate the aortic arch. We placed a 27‐gauge needle next to the arch. A 7.0 PVDF suture was threaded around the needle and aorta between the innominate and left common carotid arteries and a simple knot was tied. After confirmation of ligation, the J‐hook and excess suture were removed. The intercostal space and the skin were closed using a 5.0 resorbable suture. The isoflurane was stopped, and mice were extubated once spontaneously respiration returned. Mice were allowed to recover under a warming heat pad and were observed further throughout the perioperative period. Following the recovery period, mice were injected twice a day with buprenorphine (1 mg/kg, s.c.).

### 4.3 Ultrasound imaging

The Vevo 3100 high‐frequency ultrasound system (FUJIFILM VisualSonics) with a 40‐MHz center frequency MX550D linear array probe was used to acquire high‐resolution 2D and 4D data of the left ventricle and left atrium. We anesthetized the animals as described above without intubation. We removed the ventral thorax hair and secured the mice to the imaging stage in a supine position. The ECG and respiratory rate were monitored throughout the imaging process.

Two‐dimensional (2D) echocardiography was performed according to American Physiological Society guidelines for cardiac function measurements in mice.^35^ Left ventricular volumes and strain were analyzed in the parasternal long‐axis view with ECG‐gated kilohertz visualization (EKV) images captured at a frame rate of 1000 frames/s. We acquired four‐chamber B‐mode images at 232 frames/s to evaluate ventricular diastolic function using early diastolic strain rate, E/A ratio, and IVRT in addition to atrial function. EKV images of the left atrium and left atrial appendage were obtained in a modified short‐axis view (Figure 1B,C). The imaging stage was tilted 15 degrees to the animal's right. The ultrasound probe was positioned in a short‐axis view, on the left side of the mouse's chest, near the mid‐thoracic area, just left of the sternum and perpendicular to the chest wall. To allow for clear visualization of the LA, the probe was positioned between the LV base and the pulmonary artery. This orientation enabled us to easily detect both the LA and left atrial appendage (LAA). If both LA and LAA were not visible, the probe was fanned superiorly or inferiorly to obtain the desired view (Figure 2A).

Four‐dimensional (4D) echocardiography was performed at baseline and 4‐weeks post‐TAC. In the parasternal short axis, a linear step motor with step size 0.13 mm scanning at 400 frames/sec from the apex of the heart to the above the aortic arch was used to acquire 4D data of the left atrium. 4D data not available for all mice.

B‐mode and pulse‐wave Doppler imaging were used to confirm TAC and evaluate constriction blood flow velocity. PW Doppler imaging was performed at all time points. We conducted image analysis using Vevo Lab Software 5.9.0 and Vevo Strain 2.0 (FUJIFILM VisualSonics).

### 4.4 LA volume and strain measurement

The LA and LAA were considered one chamber for volume and strain analyses due to anatomic and functional considerations.^19^ Using Vevo Strain 2.0, we drew continuous contours along the LA endocardium from the anterior to posterior mitral valve at both maximum and minimum volumes (Figure 2A). The commercially available software then used a speckle‐tracking algorithm to trace atrial myocardial motion over the cardiac cycle and derive strain estimates. Three segmental curves were obtained throughout the cardiac cycle. The average of the peak longitudinal strain absolute values was calculated to represent the global reservoir strain. Contractile strain was also measured and defined as the second peak of atrial strain following the P wave. Conduit strain was calculated by subtracting the contractile strain from the peak reservoir strain. The software also extrapolates a volume estimate from the manually defined LA area using Simpson's method. We further validated the 2D volume by calculating 4D volumes. Using the 4D scan, we manually traced the atrial endocardium at maximum and minimum volumes in a stepwise fashion, each step 320 μm, until the entire chamber had been contoured.

### 4.5 Histology

Baseline (*n* = 4) and 4‐week TAC (*n* = 4) hearts were fixed in 4% PFA overnight, dehydrated with a sucrose gradient (final PBS 25% sucrose), frozen in OCT and sectioned (12 μm). Sections were permeabilized in PBS 0.1% triton and then blocked for 1 h in blocking solution (BS) (PBS 0.1% triton 0.1% BSA). Sections were then incubated overnight with Goat Anti‐Type I Collagen (Southern Biotech) 1/200 in BS, washed in PBS, and incubated for 2 h with secondary antibody and DAPI in BS, then washed and mounted with Fluoromount‐G (Thermo Fisher Scientific). Sections were imaged using a slide scanner (Zeiss Axioscan) and type I collagen staining was quantified in atria using ImageJ software. At least two equivalent sections of left atria were analyzed per mouse and per condition.

### 4.6 Statistical analysis

All statistical analysis was performed using Prism 10.2.3 (GraphPad Software). The threshold for statistical significance was set at *p* < 0.05. Baseline, 3‐day, 2‐, and 4‐week post‐TAC parameters are shown as mean ± standard deviation (SD). A one‐way repeated measures ANOVA with Tukey post hoc multiple pairwise comparison's test was used to compare parameters across timepoints, if unequal sample sizes, a mixed‐effects model was fit. Correlation between parameters was described using Pearson correlation coefficients if data was normally distributed as indicated by a Shapiro–Wilk test; otherwise, a Spearman's correlation coefficient and associated *p*‐value were calculated.

We performed a receiver operating characteristic (ROC) analysis to evaluate discriminative performance of left atrial function parameters on left ventricular function in the subgroup. The ROC groups were determined based on the median LV ejection fraction. LA max volume, LA ejection fraction, and LA strain were then used to discriminate between LV ejection fraction values above or below the median. All material submitted conforms to good publishing practice in physiology.^36^

## 5 CONCLUSION

Our study highlights the potential of murine LA strain analysis as a valuable tool for the early detection of LV dysfunction, offering a promising biomarker for heart failure development in pressure overload murine models. Future research should focus on the molecular mechanisms underlying these structural and functional alterations in the LA to further refine risk stratification and improve heart failure management.

## AUTHOR CONTRIBUTIONS

**John P. Salvas:** Formal analysis; investigation; visualization; writing – review and editing; writing – original draft. **Thomas Moore‐Morris:** Conceptualization; investigation; methodology; supervision; writing – original draft; writing – review and editing. **Craig J. Goergen:** Conceptualization; methodology; supervision; writing – review and editing; writing – original draft; funding acquisition; validation; investigation. **Pierre Sicard:** Conceptualization; investigation; methodology; supervision; writing – review and editing; writing – original draft; funding acquisition; validation.

## FUNDING INFORMATION

Indiana University School of Medicine (JPS), Fulbright United States Scholar Program (CJG), Thomas Jefferson Fund from the French‐American Cultural Exchange Foundation (CJG, PS), Biocampus (PS), I‐Site Muse (PS), France Life Imaging: grant ANR‐11‐INBS‐0006 (PS).

## CONFLICT OF INTEREST STATEMENT

All authors have read the journal's policy on disclosure of potential conflicts of interest. C. J. Goergen serves on the Scientific Advisory Board for FUJIFILM VisualSonics Inc. None of the other authors have conflicts of interest, financial or otherwise, to disclose. Disclaimers: FUJIFILM VisualSonics Inc. had no role in the study's design, execution, interpretation, or writing.

## Supporting information


Data S1.

## Data Availability

The data that support the findings of this study are available from the corresponding author upon reasonable request.
