# Supplementary material for: Left atrial reservoir strain as a predictor of cardiac dysfunction in a murine model of pressure overload
Source: Acta Physiol (Oxf). 2025 Jan 16;241(2):e14277. doi: 10.1111/apha.14277 (PMC11737473; doi:10.1111/apha.14277)
Supplement: Supplementary file 1 — Data S1. [file APHA-241-e14277-s001.docx]

**Supplemental Materials**

**Methods:**

Heart rate was measured during ultrasound acquisition. Cardiac output was calculated from heart rate multiplied by stroke volume from parasternal long-axis images of the left ventricle.

All statistical analysis was performed using Prism 10.2.3 (GraphPad Software). The threshold for statistical significance was set at *p* < 0.05. Baseline, 3 days, 2- and 4-weeks post-TAC parameters are shown as mean ± standard deviation (SD). A one-way repeated measures ANOVA with Tukey post-hoc multiple pairwise comparison’s test was used to compare parameters across timepoints, if unequal sample sizes, a mixed-effects model was fit. Correlation between parameters was described using Pearson correlation coefficients if data was normally distributed as indicated by a Shapiro – Wilk test; otherwise, a Spearman’s correlation coefficient and associated *p*-value were calculated.

We performed a receiver operating characteristic (ROC) analysis to evaluate discriminative performance of left atrial function parameters on left ventricular function in the main cohort. The ROC groups were determined based on the median LV ejection fraction. LA max volume, LA ejection fraction, and LA strain were then used to discriminate between LV ejection fraction values above or below the median.

A principal component analysis (PCA) was performed to elucidate meaningful variables. The analysis included LA and LV function parameters from baseline, 2-, and 4-weeks post TAC. A standardized PCA method was used to account for variable scale differences. Principal components (PCs) were selected based on a parallel analysis with 1,000 simulations and a 95% percentile level. The first two PCs are plotted to visualize how PCA discriminates between groups of LV ejection fraction above or below the median given all the data. The contribution of the variables to PC1 is shown to evaluate variable importance.

**RESULTS**

**Left Atrial Contractile and Conduit Strain**

In the main cohort, LA contractile strain was 13.7 ± 5.8% at baseline and significantly decrease to 4.7 ± 2.7% at 2-weeks post-TAC (*p*<0.001). LA contractile strain remained decreased at 6.8 ± 5.5% at 4-weeks post-TAC. Conduit strain was 7.2 ± 3.4% at baseline and decreased to 3.5 ± 2.4% at 2-weeks post-TAC (*p*=0.002) and was consistently decreased at 3.4 ± 2.7% at 4-weeks post-TAC (Supplemental Figure 1A-C)

In the subgroup, LA contractile strain was 14.4 ± 5.8% at baseline and significantly decrease to 3.1 ± 1.6% at 2-weeks post-TAC (*p*<0.001). LA contractile strain remained decreased at 4.7 ± 4.8% at 4-weeks post-TAC. Conduit strain was 11.4 ± 6.6% at baseline and decreased to 2.8 ± 2.2% at 2-weeks post-TAC (*p*=0.002) and was consistently decreased at 1.6 ± 2.7% at 4-weeks post-TAC (Supplemental Figure 1D-E).

**Heart Rate and Cardiac Output**

In the main cohort, the heart rate was 502 ± 49 bpm at baseline, 487 ± 41 bpm at 2-weeks, and 521 ± 45 at 4-weeks. Although there was a statistical difference (*p*=0.011) between 2- and 4-weeks, we found no correlation between left atrial function (r=-0.072, *p*=0.626) or left ventricular (r=-0.226, *p*=0.123) function and heart rate. Furthermore, the heart rate range was 407 to 583 bpm, all of which fall within the acceptable guideline recommended range (Supplemental Figure 2A-C).

In the subgroup, the heart rate was 531 ± 48 bpm at baseline, 484 ± 39 bpm at 3-days, and 536 ± 36 at 4-weeks. Although the heart rate at 3-days was significantly different than baseline (*p*=0.012) and 4-weeks (*p*=0.034), we found no correlation between left atrial (r=0.034, *p*=0.857) or left ventricular (r=-0.267, *p*=0.154) function and heart rate. The heart rate range was 435 to 584 bpm, and all of which are within the acceptable range (Supplemental Figure 2D-F).

The cardiac output was 14.07 ± 2.97 mL/min at baseline and decreased to 10.47 ± 3.80 mL/min 3-days post-TAC (p=0.023). The cardiac output was 12.22 ± 2.34 mL/min at 2-weeks post-TAC (*p*=0.282 vs. baseline) and 11.40 ± 1.88 mL/min at 4-weeks post-TAC (*p*=0.011 vs. baseline; Supplemental Figure 2G). We previously correlated LA function with other surrogate measurements of heart failure such as LV ejection fraction and strain. To further validate this finding, we correlated LA longitudinal strain with the cardiac output. The Spearman correlation is presented in Supplemental Figure 2H and showed r=0.331 with *p*=0.006 further suggesting LA dysfunction is a predictor of heart failure.

**ROC and Principal Component Analyses**

Additionally, we further tested that LA dysfunction could serve as a useful imaging biomarker by discriminating between low and high LV EF groups, based on the median LV EF of 53.7%. The ROC curves illustrate the diagnostic performance of LA max volume (AUC=0.858), LA EF (AUC=0.901), and LA longitudinal strain ~~(AUC=0.878)~~ (AUC=0.890) in differentiating between TAC mice with varying levels of LV EF in the main cohort. All parameters of LA function were able to significantly discriminate between low and high LVEF with *p*<0.0001 ~~(Figure 4D)~~ (Supplemental Figure 5A).

We then used principal component analysis (PCA) to highlight variables accounting for the inherent variability in the data. The first two principal components (PCs) explained ~~64.7%~~ 64.9% of the variance and visually discriminated between low and high LV EF groups ~~(Figure 4E-F)~~ (Figure (Supplemental Figure 5B-D). Interestingly, LA structural and functional modifications contributed ~~64.61%~~ 64.98% to the weight of the PCs.

**Supplemental Figures:**


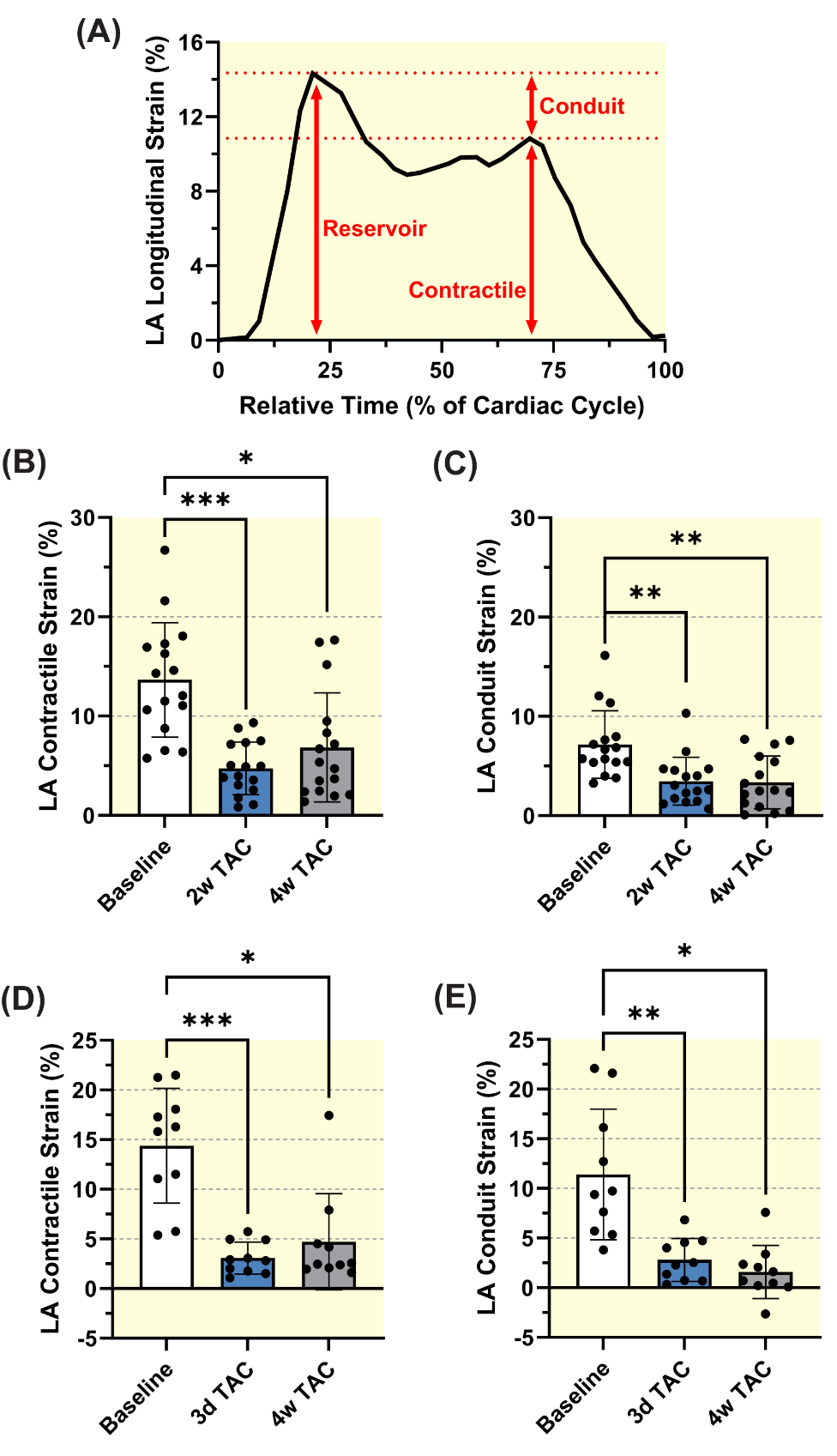


**Supplemental Figure 1:** Left atrial (LA) contractile and conduit strains. **(A)** Representative baseline LA longitudinal strain curve over the cardiac cycle with definitions of reservoir, contractile, and conduit strains. LA **(B)** contractile and **(C)** conduit strains in the main cohort. Sample size n=16 for each time points. Results expressed as mean ± SD. Statistical comparisons conducted with one-way repeated measures ANOVA with Tukey’s post hoc multiple comparisons tests. LA **(D)** contractile and **(E)** conduits strains in the cohort. Sample size n=10 for baseline and 4-weeks TAC, and n=9 at 3-days due to difficulty imaging. Results expressed as mean ± SD. Statistical comparisons conducted with mixed effects model with Tukey’s post host multiple comparisons tests to account for missing data.


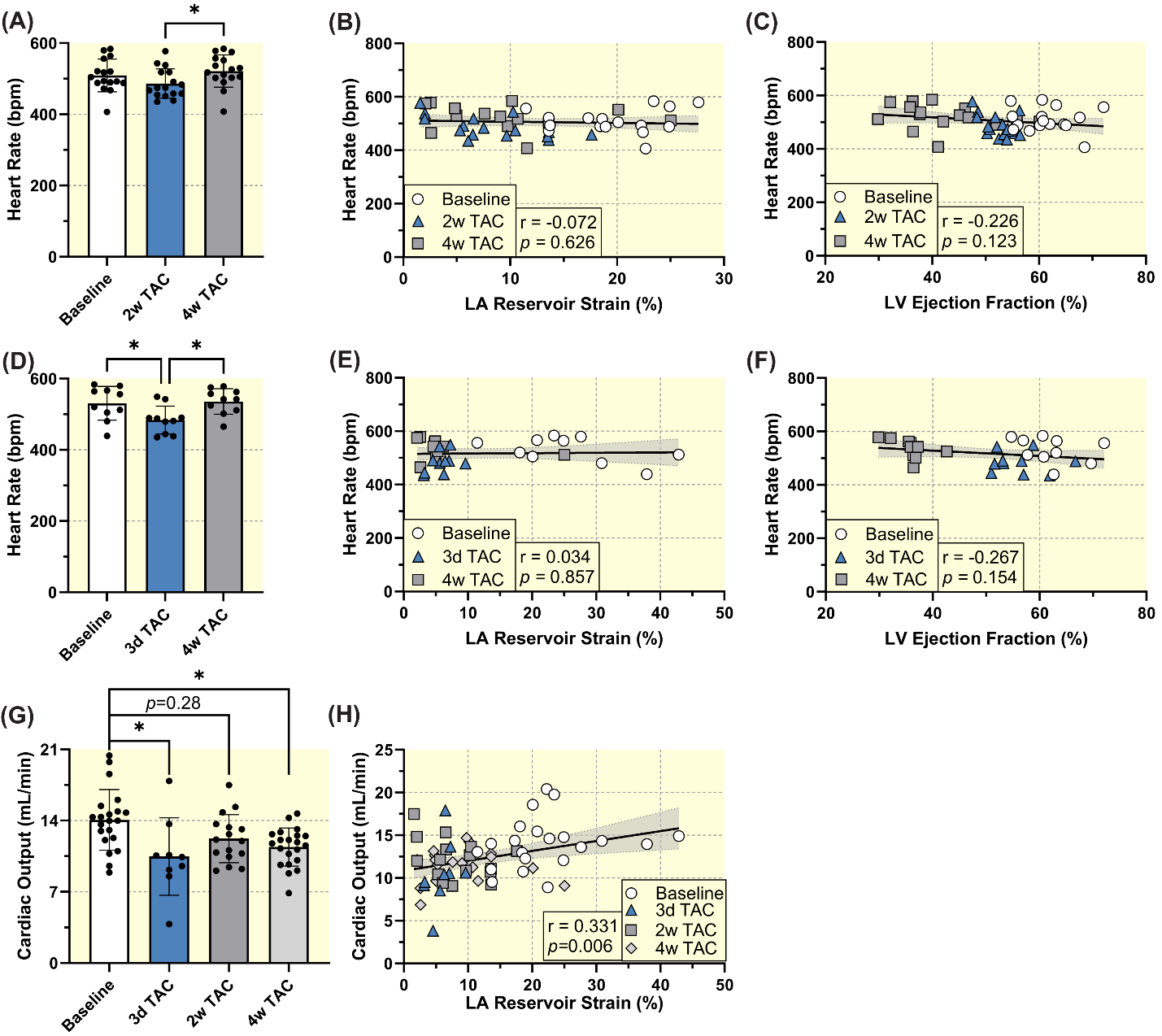


**Supplemental Figure 2:** Heart rate and Cardiac Output. **(A)** Heart rate in the main cohort. Sample size n=16 for each timepoint. Pearson correlation of heart rate with **(B)** left atrial (LA) reservoir strain and **(C)** left ventricular (LV) ejection fraction from all timepoints in the main cohort. Sample size n=48. Correlation coefficients and corresponding *p*-values displayed. **(D)** Heart rate in the subgroup. Sample size n=10 for each timepoint. Pearson correlation of heart rate with **(E)** LA reservoir strain and **(F)** LV ejection fraction from all timepoints in the subgroup. Sample size n=30. Correlation coefficients and corresponding *p*-values displayed. **(G)** Cardiac output from parasternal long-axis images. Sample size n=21, 9, 16, and 21 at baseline, 3d TAC, 2w TAC, and 4w TAC, respectively. **(H)** Spearman correlation of cardiac output with LA reservoir strain from all timepoints with data. Sample size n=67. Correlation coefficient and corresponding *p*-value displayed. Statistical comparisons in **A** and **D** conducted with one-way repeated measures ANOVA with Tukey’s post hoc multiple comparisons tests. Mixed effects model fitted in **G** to account for variable sample sizes.


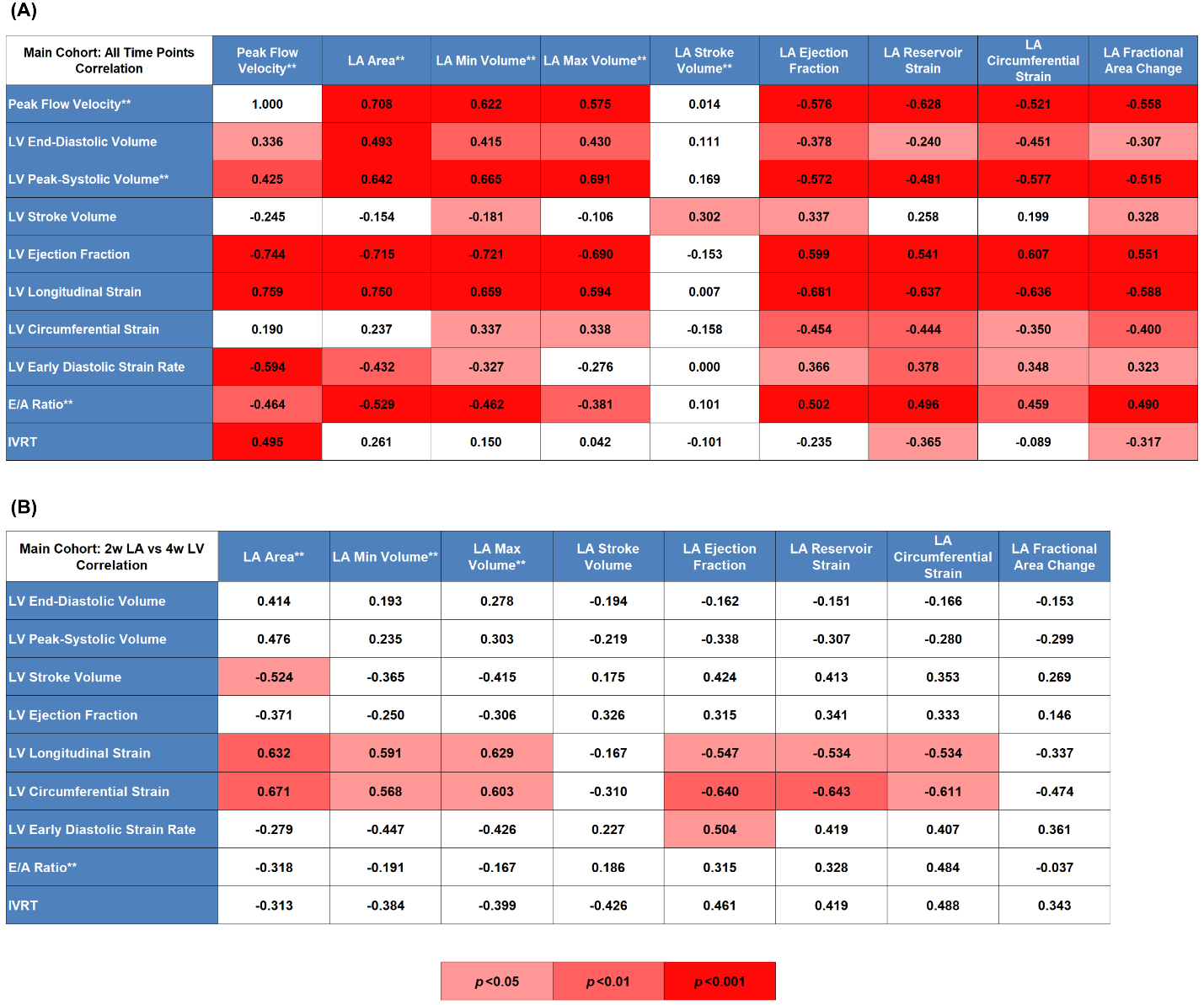


**Supplemental Figure 3:** Correlation of left atrial and left ventricular function parameters in the main cohort. **(A)** Correlation between left atrial and left ventricular function parameters from all time points, n=48. **(B)** Correlation between left atrial function parameters at 2 weeks post-TAC and left ventricular function parameters at 4 weeks post-TAC, n=16. Pearson correlation coefficients, or Spearman coefficients if data non-normally distributed as determined by Shapiro-Wilk test, displayed. Colors depict levels of statistical significance from corresponding *p*-values. **Indicates a Spearman correlation was conducted for the entire row or column.


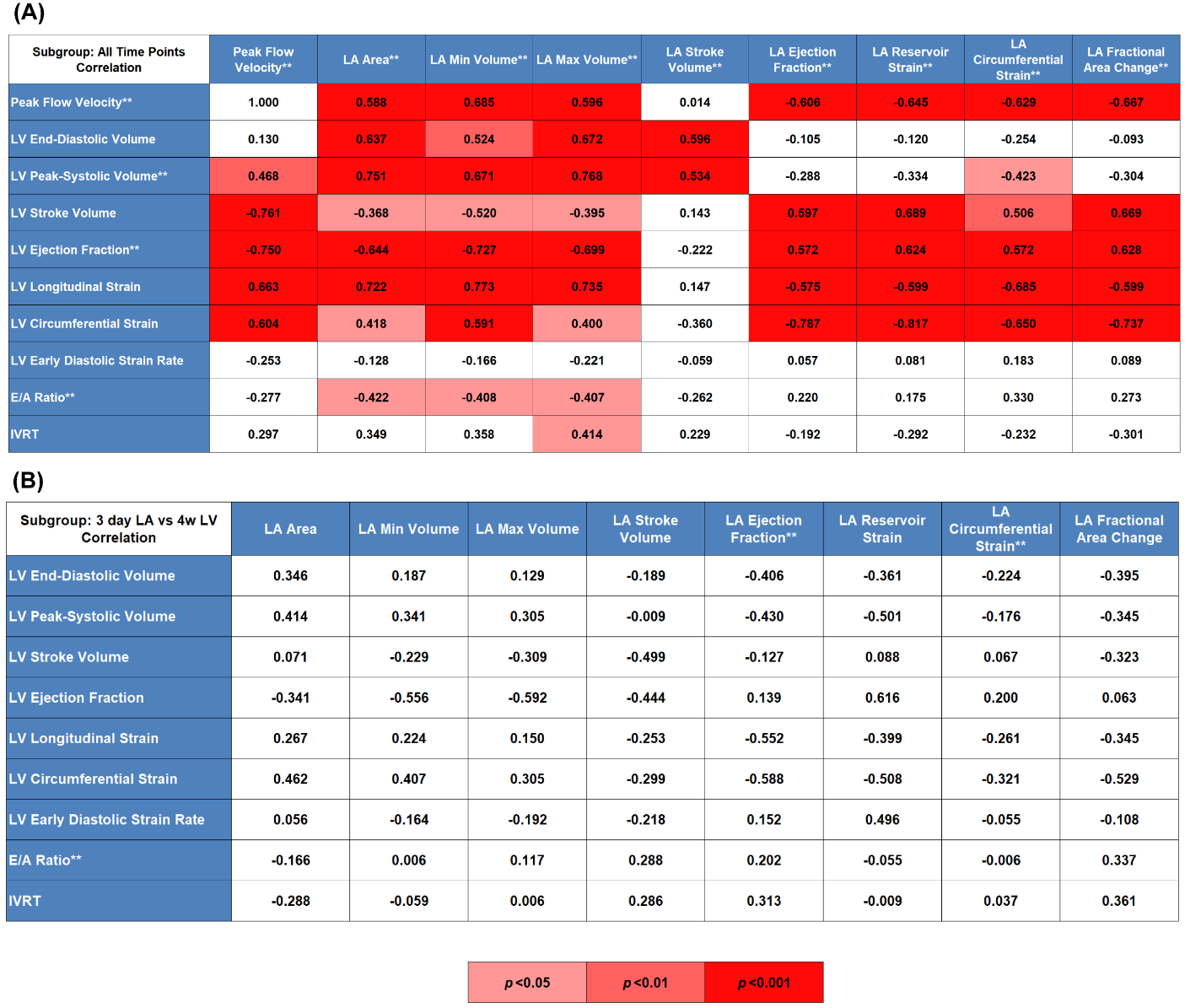


**Supplemental Figure 4:** Correlation of left atrial and left ventricular function parameters in the subgroup. **(A)** Correlation between left atrial and left ventricular function parameters from all time points, n=30. **(B)** Correlation between left atrial function parameters at 2 weeks post-TAC and left ventricular function parameters at 4 weeks post-TAC, n=10. Early diastolic strain rate n=8, E/A ratio and IVRT n=9 due to imaging challenges. Pearson correlation coefficients, or Spearman coefficients if data non-normally distributed as determined by Shapiro-Wilk test, displayed. Colors depict levels of statistical significance from corresponding *p*-values. **Indicates a Spearman correlation was conducted for the entire row or column.


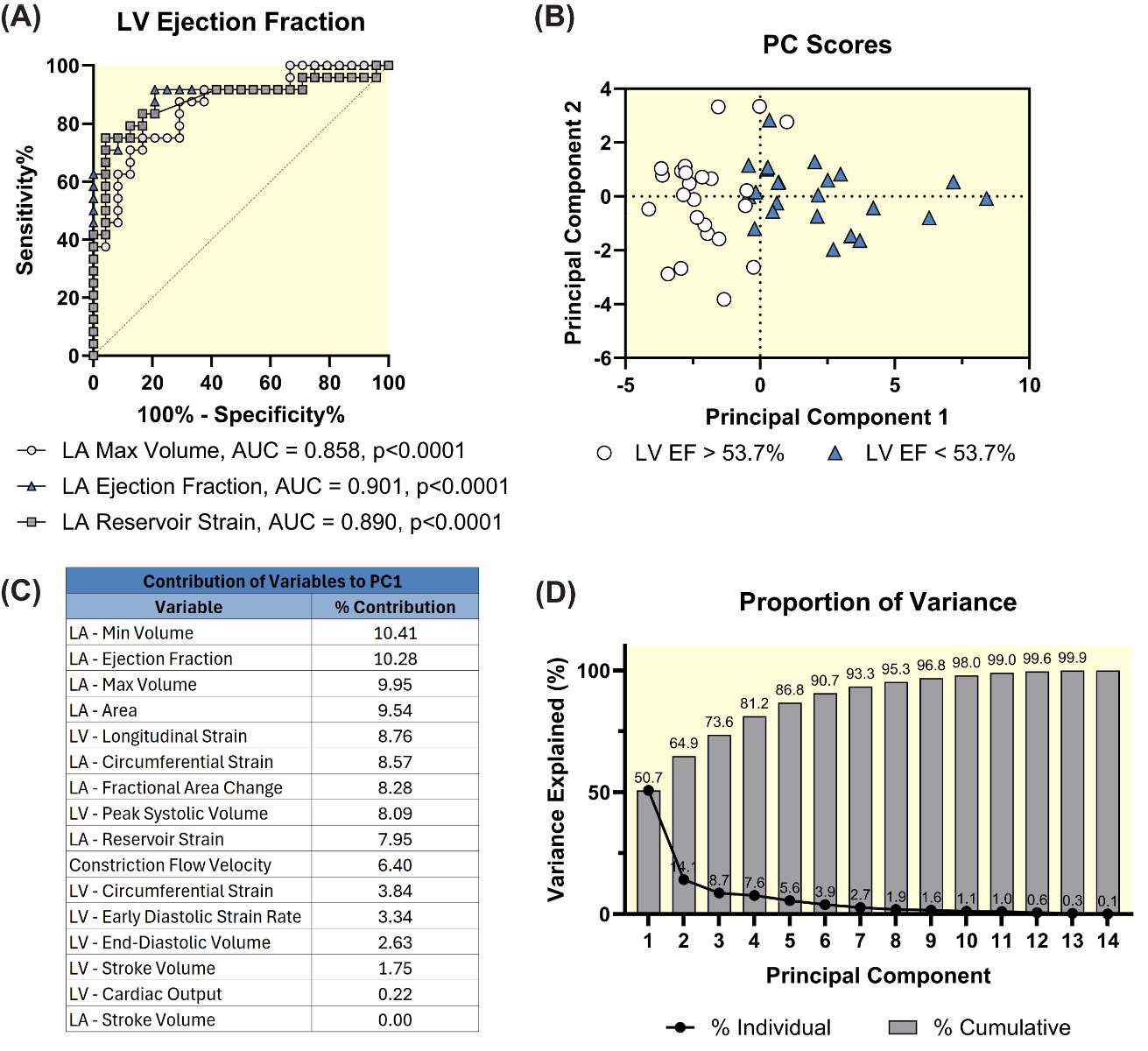


**Supplemental Figure 5:** Early left atrial dysfunction predicts ventricular dysfunction. **(A)** ROC analysis using left atrial parameters (max volume: AUC=0.858, *p*<0.0001; ejection fraction: AUC=0.901, p<0.0001; reservoir ~~longitudinal~~ strain: ~~AUC=0.878~~ AUC=0.890, *p*<0.0001) to predict ventricular ejection fraction above (n=24) or below (n=24) the median (LVEF = 53.7%). **(B)** Principal component analysis scores plot separated by left ventricular ejection fraction above or below the median. **(C)** Ranking of variables in the principal component analysis which contribute most to the separation of groups. **(D)** Proportion of variance in LV ejection fraction accounted for by each successive principal component.
